# The NR4A2 Nuclear Receptor Is Recruited to Novel Nuclear Foci in Response to UV Irradiation and Participates in Nucleotide Excision Repair

**DOI:** 10.1371/journal.pone.0078075

**Published:** 2013-11-06

**Authors:** Kasturee Jagirdar, Kelvin Yin, Matthew Harrison, Wen Lim, George E. O. Muscat, Richard A. Sturm, Aaron G. Smith

**Affiliations:** 1 Institute for Molecular Bioscience, The University of Queensland, Brisbane, Queensland, Australia; 2 School of Biomedical Science, The University of Queensland, Brisbane, Queensland, Australia; Universita’ di Milano, Italy

## Abstract

Ultraviolet radiation (UVR) is one of the most common mutagens encountered by humans and induces the formation of cyclobutane pyrimidine dimers (CPDs) and pyrimidine-(6-4)-pyrimidone photoproduct (6-4PP) lesions in the genomic DNA. To prevent the accumulation of deleterious mutations these lesions must be efficiently repaired, primarily by nucleotide excision repair. We have previously demonstrated that the NR4A family of nuclear receptors are crucial mediators of the DNA repair function of the MC1R signalling pathway in melanocytes. Here we explore the role of the NR4A2 protein in the DNA repair process further. Using EYFP tagged-NR4A2 we have demonstrated a UVR induced recruitment to distinct nuclear foci where they co-localise with known DNA repair proteins. We reveal that the N-terminal domain of the receptor is required for this translocation and identify a role for p38 and PARP signalling in this process. Moreover disruption of the functional integrity of the Ligand Binding Domain of the receptor by deleting the terminal helix 12 effectively blocks co-localisation of the receptor with DNA repair factors. Restored co-localisation of the mutant receptor with DNA repair proteins in the presence of a Histone Deacetylase Inhibitor suggests that impaired chromatin accessibility underpins the mis-localisation observed. Finally NR4A2 over-expression facilitated a more efficient clearance of UVR induced CPD and 6-4PP lesions. Taken together these data uncover a novel role for the NR4A nuclear receptors as direct facilitators of nucleotide excision repair.

## Introduction

The NR4A transcriptional regulators are a 3 member orphan sub-family of Nuclear Hormone Receptors that includes NR4A1 (Nur77/NGFI-B), NR4A2 (Nurr1, NOT) and NR4A3 (NOR-1, MINOR). NR4A proteins contain the highly conserved zinc-finger DNA binding domain (DBD) and carboxy-terminal ligand binding domain (LBD) characteristic of nuclear receptors [1], [2]. The amino terminal domain of the receptor is less conserved and the functional importance of this region of the receptor remains poorly understood. The NR4A receptors have been found to regulate gene expression programs that govern a wide range of biological processes ranging from cell proliferation, differentiation and development to metabolism, inflammation and vascular disease [1], [2]. While no endogenous ligands have been identified for these receptors, and the crystal structure of the LBD suggests they are true orphan receptors, NR4A proteins have been found to be tightly regulated by numerous signalling pathways at both the transcriptional and post-translational level allowing these proteins to function rapidly and specifically to co-ordinate diverse cellular responses. We have previously reported the rapid induction of the NR4A family by the Melanocortin-1 Receptor (MC1R) in melanocytes [3]. MC1R is a G-protein coupled receptor that is a central regulator of pigmentation in melanocytes and has also been shown to facilitate DNA repair and cytoprotection following exposure to ultraviolet UV radiation (UVR). Our studies demonstrated that the NR4A1 and NR4A2 proteins are required for the ability of MC1R to enhance DNA repair following UVR [3].

Maintenance of an organism’s cellular genome integrity following a mutagenic insult is vital to prevent deleterious consequences such as cell death and cancer. Exposure to ultraviolet radiation (UVR), one of the most commonly encountered exogenous carcinogens for humans, results primarily in the formation of cyclobutane pyrimidine dimer (CPD) and 6-4-photoproduct (6-4PP) lesions in the DNA [4]. Failure to adequately identify and repair such lesions ultimately results in the formation of cancer causing mutations that underpin the significant association between sun exposure and the development of melanoma and non-melanoma skin cancers. Accordingly, cells have evolved numerous mechanisms to deal with the DNA damage, with Nucleotide excision repair (NER) being the principle pathway responsible for the repair of UVR induced CPD and 6-4PPs. The importance of the NER pathway is exemplified by individuals with the rare genetic disorder Xeroderma Pigmentosum that possess mutations in various proteins of the NER pathway and are hypersensitive to UVR and have up to a 1000 fold greater incidence of skin cancer than the general population [5].

Nucleotide excision repair (NER), like other DNA repair mechanisms, proceeds as a highly ordered sequential process involving the recruitment of an array of factors [5], [6]. Briefly, the site of photo-lesion is recognised by the DNA damage-binding protein 1, 2 complex (DDB1-DDB2), which weakens the histone-DNA interactions [7], [8]. The XPC complex is then recruited to provide docking site for the ten-component basal transcription factor, THIIH then acts as a DNA helicase to unwind the DNA-nucleosome polymer [9]. The damaged oligonucleotides are then cleaved by XPG and XPF-ERCC1 at 3′ and 5′ ends, respectively. The repair is finally completed by the resynthesis of oligonucleotides by polymerases δ, ε or κ [10].

Our previous identification of the central role of the NR4A proteins in MC1R mediated DNA repair in melanocytes suggested an unexpected role for these receptors in the DNA repair process, however the mechanism and transcriptional targets responsible for this phenomenon remained to be identified. Accordingly we wished to explore the role of the NR4A family in the DNA repair process. Here we report the rapid recruitment of the NR4A2 protein to distinct nuclear foci in response to UVR treatment of cells. Moreover, co-localisation studies with known DNA repair proteins suggest these foci are potentially sites of active DNA repair. Using mutant NR4A2 we demonstrate the importance of the NR4A LBD in influencing the co-localisation between NR4A2 and DNA repair proteins in response to DNA damage and repair. Moreover, our data indicates that the NR4A family are key participants in the DNA repair process, independent of their actions as transcriptional regulators, potentially by influencing chromatin accessibility.

## Results

### Recruitment of the NR4A2 Protein to Nuclear Foci upon UVR Exposure

**Figure 1:**
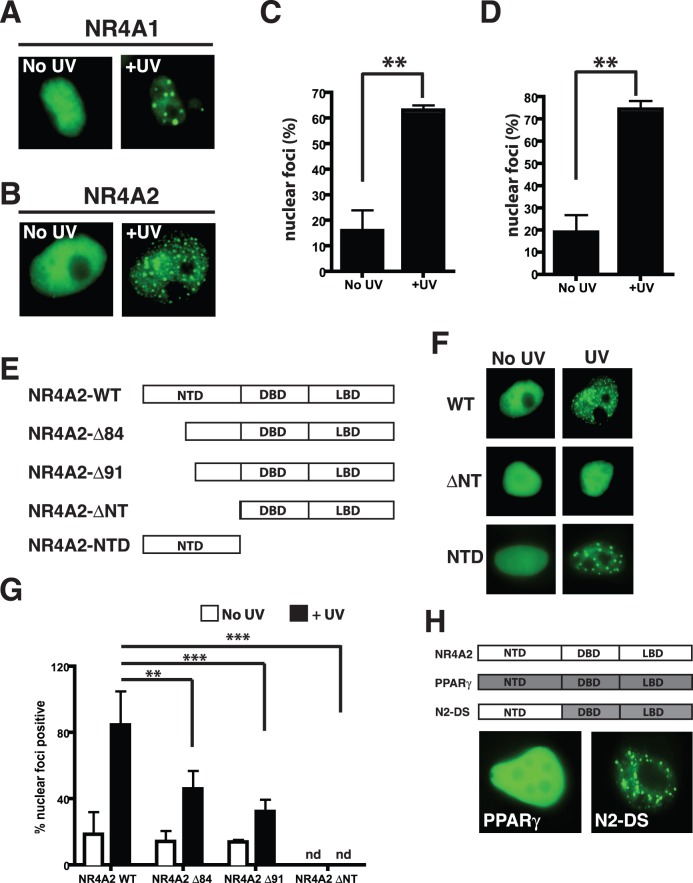
UVB irradiation induces NR4A nuclear foci formation. (**A–B**) MM96L cells transiently transfected with EYFP-NR4A1 (**A**) or EYFP-NR4A2 (**B**) were mock (No UV), or UVR (+UV) treated, and fixed 4 hours post treatment. Localisation of the NR4A fusion proteins was determined by fluorescence imaging, representative cells are shown. (**C–D**) Relative quantification of UV induced NR4A nuclear foci was performed by blind scoring images as diffuse or nuclear foci positive for NR4A1 (**C**) or NR4A2 (**D**). Data represents the pooled relative percentages from at least three independent experiments. An unpaired student t-test was used to determine the statistical differences between control and UV treated cells (**p<0.01, ***p<0.001). (**E**) A schematic representation of the NR4A2 WT, NTD alone and sequential N-terminal deletion mutants of NR4A2 used to identify the region responsible for translocation to foci. In all cases the EYFP fluorophore is fused at to the N-terminal end of the protein. (NTD, N-terminal domain; DBD, DNA binding domain; LBD, ligand binding domain). (**F**) Representative images of nuclear localisation of EYFP-WT and ΔNT and NTD NR4A2 proteins following mock or UVR treatment, as indicated. (**G**) Localisation of EYFP-NR4A2 WT, Δ84, Δ91 and ΔNT proteins to nuclear foci in response to mock (white boxes) or UV irradiation (black boxes) was scored as foci positive or negative in blind analysis. Data represents the mean +/− SEM of three independent experiments. Statistical analysis was performed using ANOVA with a Tukeys post-test (***, p<0.001; **, p<0.01, ns = not significant, nd = 0 foci positive nuclei detected). (**H**) Schematic representation of an NR4A2 N-terminal domain- PPARγ dominant negative-LBD chimera termed N2-DS. Lower panels show the post UVR localisation of EYFP tagged PPARγ and N2-DS chimera in the nucleus of transfected cells.

The NR4A1/Nur77 protein has been shown to undergo nuclear redistribution to distinct foci upon treatment of cells with DNA damaging agents [14]. In light of our previous demonstration that the NR4A proteins were pivotal to MC1R mediated DNA repair in melanocytes [3], we wished to determine if a similar NR4A1 recruitment to nuclear foci would occur in response to UV radiation and if this phenomenon would also occur for the NR4A2 protein. To examine this we transfected MM96L melanoma cells with constructs expressing NR4A1 or NR4A2 with an N-terminal EYFP fusion, after which we irradiated the cells with 25 mJ/cm^2^ of polychromatic UVA/B [3]. Following UVR cells were fixed at different time points between 0 and 6 hours post treatment and examined by fluorescent microscopy for the localisation of the NR4A fusion proteins within the nuclei. As expected both NR4A proteins exhibited a diffuse nuclear localisation in the mock treated cells, while a redistribution of the protein into distinct nuclear foci varying in size and number was most evident 4 hours post UV irradiation (Figure 1A and B). Nuclei from multiple experiments were scored as either having distinct nuclear foci or diffuse presentation at the 4 hour time-point and these data reveal a significant increase in NR4A foci following UV irradiation compared to the non-irradiated controls for both NR4A1 (Figure 1C) and NR4A2 (Figure 1D). Similar fluorescent localisation was performed using other nuclear receptor proteins including PPARγ (Figure 1H) and RXRγ (not shown) to determine whether recruitment to nuclear foci upon UV irradiation was a unique function of the NR4A family. UVR induced nuclear foci was not observed for the PPARγ or RXRγ proteins.

### The N-terminal Domain of NR4A2 is Required for Nuclear Foci Formation

To investigate this phenomenon further we had available a sequential N-terminal deletion series of the NR4A2 protein fused to EYFP (Figure 1E) and performed a similar localisation following UV irradiation. Removal of the N-terminal domain of NR4A2 resulted in complete loss of recruitment of the EYFP-NR4A2ΔNT fusion protein to nuclear foci following UV irradiation (Figure 1F), while intermediate deletions EYFP-NR4A2Δ84 and EYFP-NR4A2Δ91 retained the ability to be recruited to these sites albeit with variable efficiency (not shown). Fusion of the NTD alone to EYFP was sufficient to induce recruitment to foci in response to UV irradiation (Figure 1F). In order to quantify this recruitment, fluorescent images of nuclei from irradiated and non-irradiated cells were scored for the presence of distinct, irrefutable foci. This analysis suggests that the efficiency of recruitment to foci is reduced with each deletion although is not completely abolished until the entire N-terminal domain is removed (Figure 1G). A chimeric nuclear receptor was generated where the N-terminal domain (NTD) of a dominant negative mutant form of the PPARγ receptor [20] was replaced with the NR4A2 NTD (termed N2-DS) in order to determine if this domain was sufficient to facilitate recruitment of the receptor to nuclear foci, as the full length mutant PPARγ was unable to undergo this translocation (Figure 1H). UV irradiation of cells expressing the N2-DS chimera resulted in the accumulation of the protein in foci similar to those observed with the WT NR4A2 protein. Taken together these data demonstrate that the N-terminal domain of NR4A2 is sufficient for recruitment to nuclear foci following UV irradiation.

### p38 Signalling is Required for UV Induced NR4A2 Foci Formation

**Figure 2:**
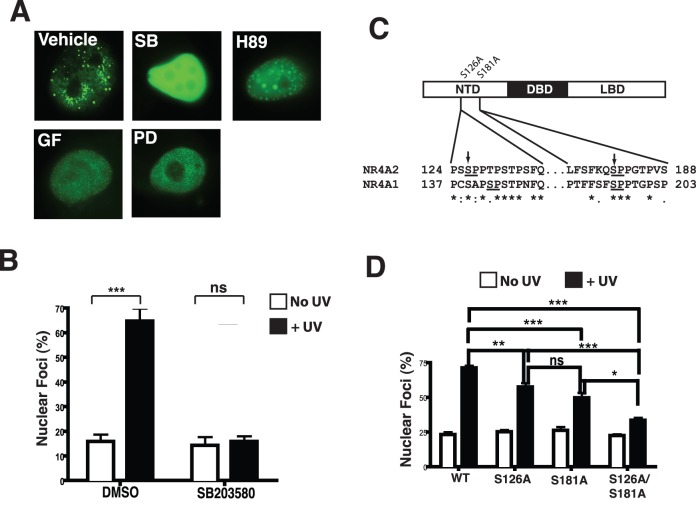
p38 signalling mediates UV induced localisation of NR4A proteins to sites of DNA damage repair. (**A**) Fluorescent images of EYFP-NR4A protein localisation in nuclei following UVR treatment in the presence of inhibitors of p38 (SB203580; SB), PKA (H89), PKC (GF109203X; GF) or MEK1/2 (PD98059; PD) or DMSO (Vehicle). Representative images from 4 independent experiments are shown. (**B**) Nuclear images were scored as diffuse or foci positive from vehicle and p38 inhibitor treatments described above in mock and UVR treated cells. Bar graph shows the mean percentage of foci positive cells (+/− SEM). (**C**) Schematic representations of NR4A2 protein showing position of potential of Alanine substitutions at p38 phosphorylation sites in the N-terminal domain at amino acids S126 and S181. Clustal alignment below shows sequences flanking the serine residues (arrow head) and phospho sites (underlined). (**D**) Scoring of foci containing nuclei in cells expression WT and S126A, S181A and S126A/S181A mutant proteins as indicated. Bar graph represents the mean +/− SEM of results from 4 independent experiments. Statistical analysis in panels (B) and D was performed using ANOVA with a Tukey’s post-test (***, p<0.0001; **, p<0.001; *, p<0.01; ns, not significant).

Similarly, we examined UV induced re-localisation of NR4A proteins following pre treatment of cells with chemical inhibitors of key signal transduction pathways including PKA (H89), PKC (GF109203X), MEK1/2 (PD98059) and p38 (SB203580). Inhibition of PKA, PKC or MEK1/2 kinases did not affect NR4A2 localisation to nuclear foci, in response to UV irradiation, however the treatment of cells with the p38 inhibitor completely abolished the recruitment to nuclear foci (Figure 2A and B). Given the p38 pathway has been shown to directly target NR4A function [15] we speculated that NR4A2 foci might be regulated through direct phosphorylation of the N-terminal domain by p38. Hence, to determine whether p38 regulates the change in NR4A2 localisation in response to UVR, we scanned the N-terminal domains of both NR4A1 and NR4A2 proteins for potential p38 consensus phosphorylation sites using GPS2.1 software [16]. Further refinement of our targeting was performed by Clustal-W alignment of the N-terminal domains of these receptors to identify potential p38 sites with conserved amino acid residues surrounding the phospho site which may suggest a docking site for protein-protein interactions that mediate the recruitment of these proteins to the observed foci. From this combined analysis two serine residues in NR4A2 at positions 126 and 181 were selected for further investigation based on significant amino acid sequence conservation in flanking residues in all three NR4A family members (Figure 2C). We generated Alanine substitution mutations in the NR4A2 NTD at S126A and S181A, as well as a double mutant form carrying both substitutions S126/181A.

Similar to our analysis of the NR4A2 N-terminal truncation mutants the Serine mutants exhibited some level of foci formation in response to UV irradiation, however these foci were variable in size and number, and more distinct foci were evident in cells transfected with wild-type NR4A2. Furthermore, quantification of the fluorescence imaging of these cells from four independent experiments revealed that mutations in the p38 consensus phosphorylation sites significantly attenuated the percentage nuclear foci formation (Figure 2D). Taken together, these observations suggest that residues, S126 and S181 are potential phosphorylation sites and are required for efficient recruitment of the NR4A2 protein to UV induced nuclear foci. These data suggest that the UVR induced translocation of NR4A2 to these foci is induced by a combination of phosphorylation at multiple residues in the N-terminal domain. Moreover, it is possible that p38-mediated translocation of these NR4A2 to foci might represent a novel pathway to DNA repair in response to DNA damage.

### NR4A2 Proteins Co-localize with DNA Repair Proteins in UV Induced Nuclear Foci

**Figure 3:**
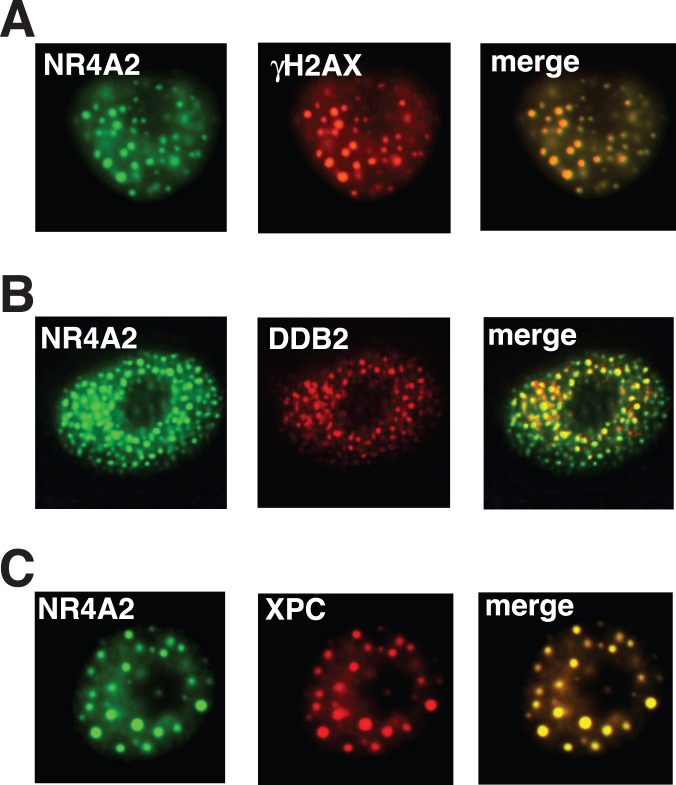
NR4A2 co-localises with DNA repair proteins following UVR treatment. MM96L cells transfected with pEYFP-NR4A2 were irradiated with 25 mJ/cm2 UV, fixed at 4 hours post-UV treatment and probed with primary antibodies against endogenous (**A**) γH2A.X or (**B**) DDB2 or (**C**) XPC proteins as indicated. Left panels, pEYFP-Nurr1 fluorescence (green); middle panel, immunofluorescence (red); centre right, merged images; right, DAPI staining. These data are representative images from 4 independent experiments.

Having established that UV irradiation induces NR4A recruitment to distinct nuclear foci, we chose to investigate whether the ensuing foci co-localised with proteins involved in DNA damage and repair. To address this question we examined the localisation of γH2A.X, a known marker of double stranded breaks which has also been reported to form foci in response to UVR, and two proteins involved in nucleotide excision repair, XPC and DDB2 [17]–[19]. To determine if these DNA repair proteins co-localize with NR4A2 in response to UVR, we transiently co-transfected the EYFP-NR4A2 expression construct into MM96L cells after which they were irradiated with 25 mJ/cm^2^ of UVR. Immuno-fluorescent detection of the γH2A.X protein in UV irradiated cells confirmed co-localisation with the NR4A2 protein (Figure 3A). Similar co-localisation of NR4A2 with γH2A.X following γ-irradiation has recently been reported and confirms a role for the NR4A proteins in the repair of double stranded breaks in the genome [20]. As the majority of photo-lesions following UV exposure are repaired by NER we wished to examine the localisation of the NR4A2 protein with components of the NER signalling pathway. Co-localisation of transfected EYFP-NR4A2 fusion proteins with endogenous DDB2 and XPC is shown in Figure 3B and 3C respectively, and suggests a similar role for NR4A2 in the process of NER. Notably, co-localisation with γH2A.X, XPC and DDB2 were not observed in cells without UV irradiation (data not shown). Taken together, these data suggest that the NR4A2 foci represent sites of DNA damage and repair.

### Disruption of the NR4A2 Ligand Binding Domain Disrupts Co-localisation with DNA Repair Proteins at DNA Damage Foci

**Figure 4:**
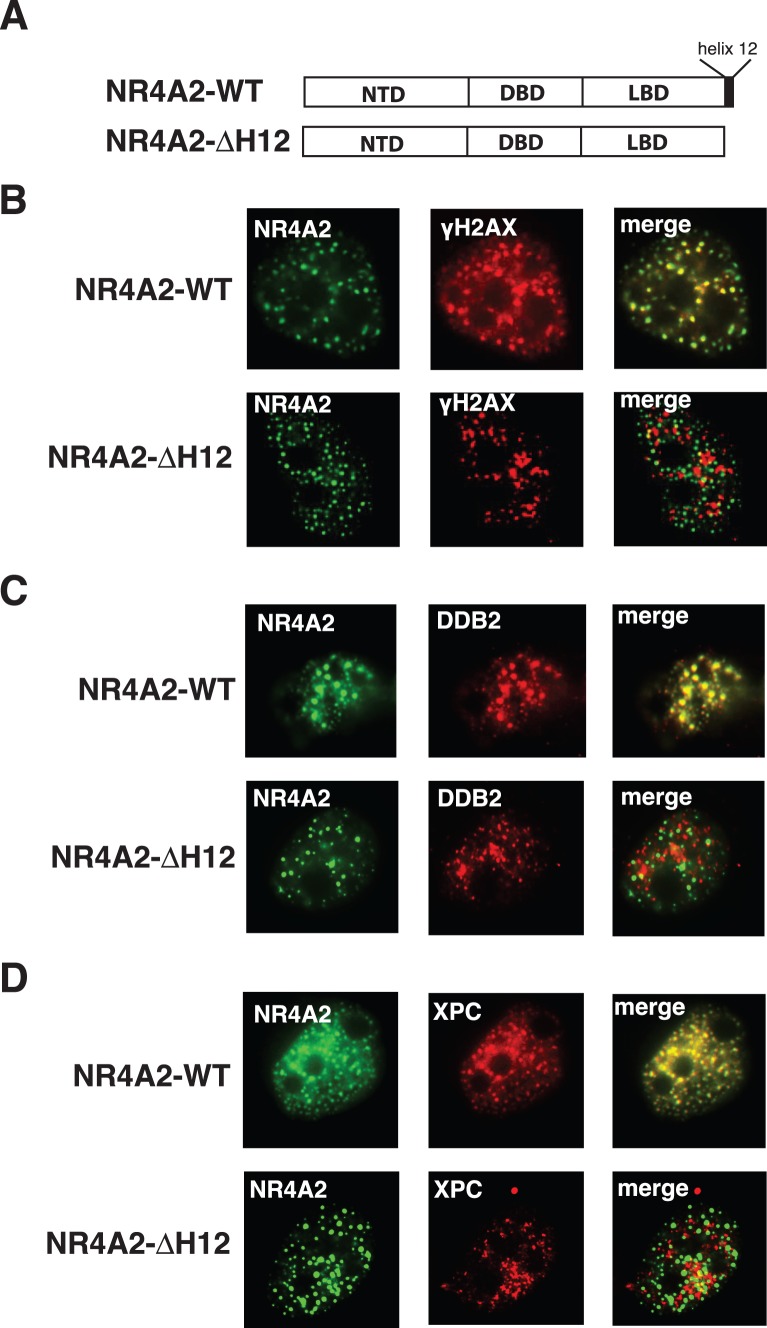
Mutations in the NR4A2 LBD result in mis-localisation of the receptor with DNA repair proteins. (**A**) Schematic representation of the WT and ΔH12 mutant forms of NR4A2 indicating the position of the C-terminal helix 12. MM96L cells transfected with WT or ΔH12 forms of NR4A2 were treated with 25 mJ/cm2 UV, fixed at 4 hours post-UV treatment. Immunofluorescence was performed to detect (**B**) γH2A.X; (**C**) DDB2; and (**D**) XPC proteins. Left panels (green fluorescence), pEYFP-NR4A-WT (upper) or pEYFP-NR4A2-ΔH12 (lower); Middle panels, γH2A.X, DDB2 or XPC immunofluorescence (red); Right panels, merged images; Representative images from 4 independent experiments is shown.

The important role of NRs in modulating chromatin environment via the recruitment of co-regulator proteins is well established in the context of transcriptional regulation. Accordingly, it is plausible that recruitment of NR4A proteins to sites of DNA damage may likewise facilitate chromatin accessibility to allow the efficient assembly of repair complexes. In order to examine the effect of NR4A2 function on the composition of the observed foci, we targeted the AF-2 region of the NR4A2 ligand binding domain (LBD) by generating a truncated form of the receptor (ΔH12) in which the C-terminal helix 12 region has been deleted (Figure 4A). The AF-2 domain of nuclear receptors is crucial for the interaction with co-repressor and co-activator proteins, which ultimately modulate chromatin architecture at the site [21], [22]. WT and ΔH12 mutant forms of NR4A2 were expressed in MM96L cells as EYFP fusions after which the cells were irradiated with UVR. As previously observed the WT NR4A2 protein was recruited to distinct nuclear foci in response to UV irradiation, which co-localised with endogenous γH2A.X, DDB2 and XPC (Figure 4B–D, upper panels). Conversely, the deletion of helix 12 in the ΔH12 mutant form resulted in a striking loss of co-localisation of the NR4A2 protein and the respective DNA repair proteins (Figure 4B–D, lower panels). While both NR4A-ΔH12 and the DNA repair proteins, γH2A.X, DDB2 and XPC, translocated to nuclear foci in response to UV induced DNA damage the proteins were rarely, if ever, co-localised together. In addition to the ΔH12 mutant, the N2-DS chimeric protein, in which the NR4A2 NTD has replaced the natural NTD of a dominant negative mutant form of the PPARγ nuclear receptor (Figure 1H), was also used in identical analysis. Hence the N2-DS chimera can be recruited to foci in response to UVR by virtue of the NR4A2 NTD, but carries with it a mutated PPARγ LBD, previously shown to function as a dominant negative receptor due to aberrant recruitment of co-repressor proteins [23]. As neither the full length WT or dominant negative mutant PPARγ proteins translocate to nuclear foci in response to UV irradiation (Figure 1H), the UV induced localisation of the N2-DS protein is attributable to the function of the NR4A2 NTD domain. Localisation of the chimeric N2-DS was observed in response to UV, however a striking mis-localisation with the DNA repair proteins was also observed similar to the ΔH12 mutant (Figure S1). Taken together these data demonstrate that the function of the NR4A2 LBD can influence the UVR induced co-localisation with DNA repair proteins.

#### Inhibition of histone deacetylase activity restores localisation of DNA repair proteins with the mutant NR4A proteins in nuclear foci

**Figure 5:**
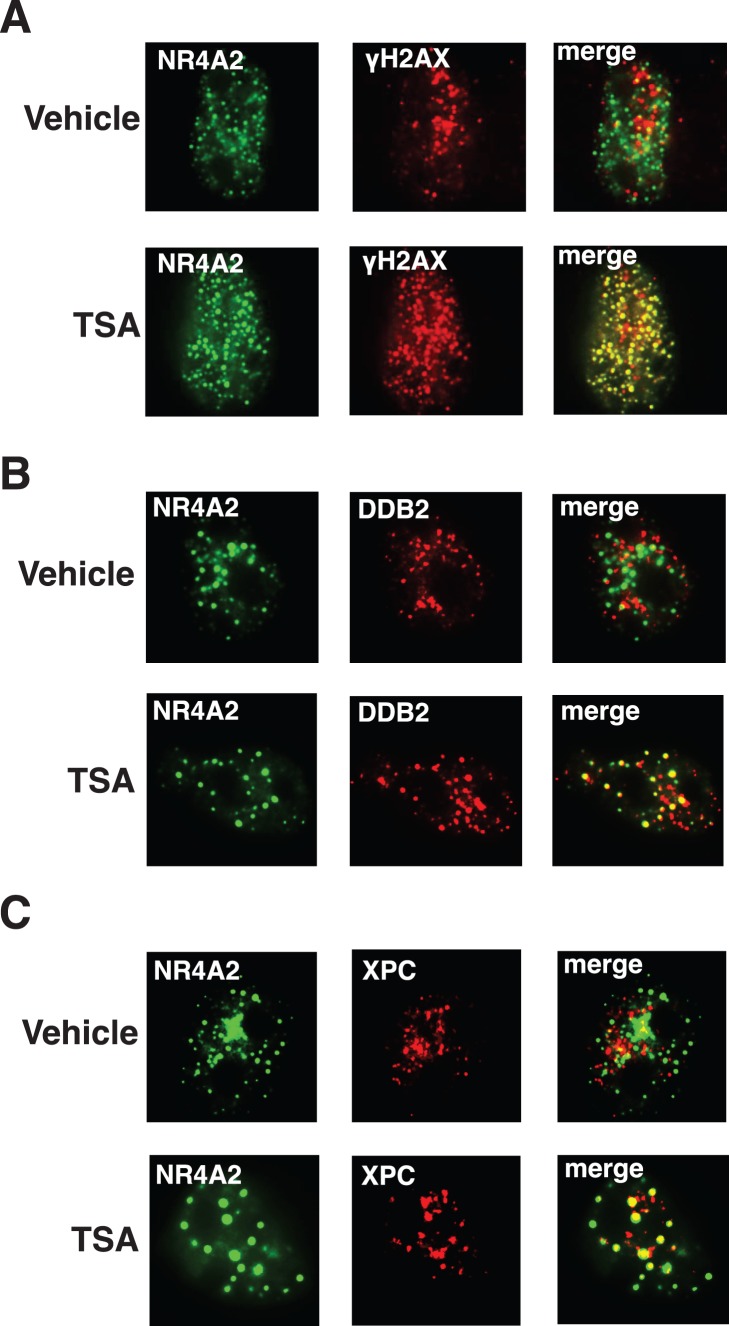
Trichostatin A (TSA) treatment restores co-localisation of NR4A2-ΔH12 and DNA repair proteins. The co-localisation of the NR4A2-ΔH12 mutant (green) and (**A**) γH2A.X, (**B**) DDB2 or (**C**) XPC shown in red was performed in the presence of vehicle (upper panels) or TSA (lower panels). These data are representative images from 4 independent experiments. Left panels (green fluorescence), pEYFP-NR4A2-ΔH12; Middle panels, γH2A.X, DB2 or XPC immunofluorescence (red); Right panels, merged images; Representative images from 4 independent experiments is shown.

As the strong interaction between the PPARγ dominant negative LBD and co-repressors has been established, it is likely that both NR4A2-ΔH12 and the N2-DS chimera, mis-localize with DNA repair proteins due to co-repressor mediated recruitment of Histone de-acetylase enzymes. In a transcriptional context such recruitment would be expected to result in the de-acetylation of the chromatin creating a closed environment inaccessible to large protein complexes [21], [22]. To test this we examined the effect of the HDAC inhibitor Trichostatin A on the mis-localisation of mutant NR4A2 and DNA repair proteins. As shown in Figure 5 the NR4A2-ΔH12 mutant did not co-localise with γH2A.X, DDB2 or XPC following UV treatment in the presence of vehicle, despite recruitment of each of the respective proteins to distinct foci in response to UVR (Figure 5A–C, upper panels). Treatment of these cells with TSA however resulted in the detection of a large number of foci in which the mutant NR4A2 protein now co-localised with the DNA repair proteins (Figure 5A–C, lower panels). A similar restoration of localisation was observed with the N2-DS chimera following TSA treatment (Figure S2). Co-transfection of the WT and ΔH12 NR4A2 constructs with an NBRE reporter construct revealed a significantly impaired transcriptional activity of the mutant receptor that was similarly restored following TSA treatment (Figure S3). These data suggest that the mutant NR4A2 proteins prevent localisation of DNA repair proteins following UV irradiation by recruiting HDACs, which de-acetylate the chromatin surrounding DNA lesions.

### NR4A2 Shows Increased UV Induced Interaction with PARP1

**Figure 6:**
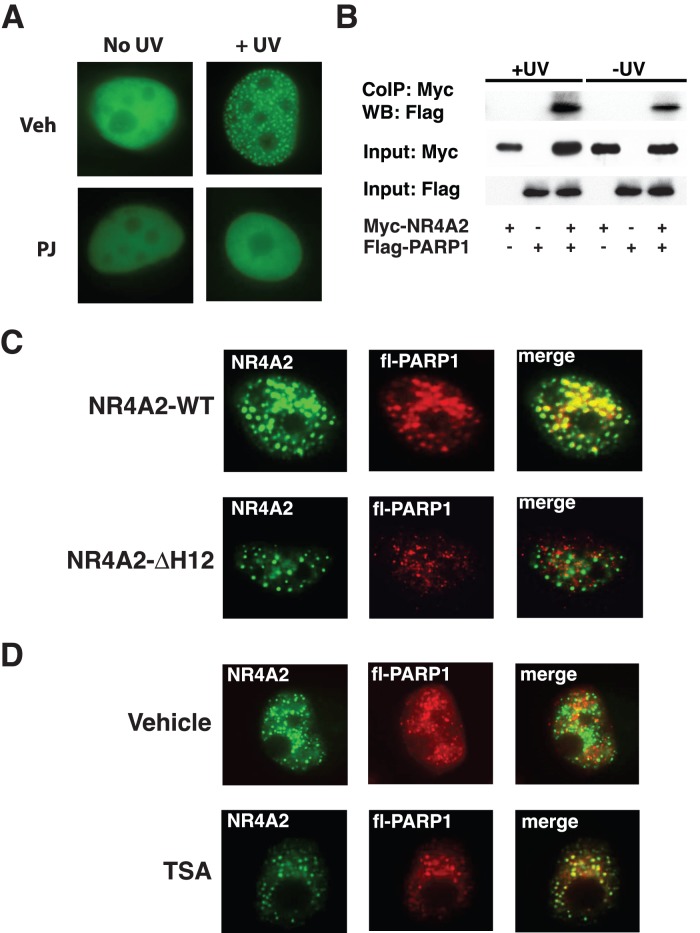
NR4A2 shows increased UV induced interaction with PARP1. (A) Fluorescence images of EYFP-NR4A2 protein localisation in nuclei following UVR treatment in the presence of inhibitor of PARP (PJ34; PJ) or Vehicle (DMSO). Representative images from 4 independent experiments are shown. (B) Co-immunoprecipitation of COS-1 cells transfected with MYC-NR4A2 and Flag-PARP1, as indicated, and mock or UV irradiated. Immunoprecipitation was performed with an anti-MYC antibody and precipitates probed with anti-FLAG antibody. A representative image taken from three independent experiments is shown. (C) MM96L cells co-transfected with WT or ΔH12 forms of NR4A2 and Flag-PARP1 (fl-PARP1) and treated with 25 mJ/cm^2^ UV, fixed at 4 hours post-UV treatment. Immunofluorescence was performed to detect FLAG-PARP1 protein. Left panels (green fluorescence), pEYFP-NR4A-WT (upper) or pEYFP-NR4A2-ΔH12 (lower); Middle panels, FLAG-PARP1 immunofluorescence (red); Right panels, merged images. (**D**) The co-localisation of the NR4A2-ΔH12 mutant (green) and FLAG-PARP1 shown in red was performed in the presence of vehicle (upper panels) or TSA (lower panels). Left panels (green fluorescence), pEYFP-NR4A2-ΔH12; Middle panels, FLAG-PARP1 immunofluorescence (red); Right panels, merged images.

Recent evidence in the context of double stranded break repair has revealed that PARP1 expression and activity is required for the recruitment of NR4A2 proteins to DSB foci [20]. Accordingly we wished to examine the effect of a small molecule inhibitor of PARP activity on the recruitment of NR4A2 to UV induced foci. As shown in Figure 6A, the co-treatment of cells with the PARP inhibitor, PJ34 (PJ) completely abolished the recruitment of the NR4A2 protein to nuclear foci following UV irradiation. As PARP1 proteins have been shown to interact directly with another member of the NR4A family, NR4A3, via the DBD [24] we sought to determine if NR4A2 could also interact physically with PARP1. Co-immunoprecipitation was performed using COS-1 cells transfected with MYC-NR4A2 and FLAG-PARP1 expression constructs after which the cells were mock treated or UV irradiated. At 4 hours post UV treatment, the protein extracts were collected and complexes precipitated with antibodies against the MYC-epitope and immunoblotted with antibodies against the FLAG-epitope (Figure 6B). This analysis revealed a basal interaction between the two proteins which appears to be enhanced following exposure of the cells to UVR (Figure 6B). Taken together, these data suggest that a direct physical interaction occurs between PARP1 and NR4A2 and that PARP1 activity is required for NR4A2 foci formation in response to UVR. To determine if PARP1 colocalizes with NR4A2 in UV induced foci a FLAG-PARP1 fusion construct was co-transfected with WT or ΔH12 mutant forms of NR4A2 and UV irradiated as previously described. Immunofluorescence using antibodies against the FLAG epitope confirmed co-localisation of PARP1 with the WT NR4A2 protein (Figure 6C, upper panels). Conversely, no co-localisation of the ΔH12 mutant and PARP1 protein was detected (Figure 6C, lower panels). As previously demonstrated with other DNA repair proteins, treatment of the cells with TSA resulted in the restoration of NR4A2-ΔH12 co-localisation with PARP1 compared to cells treated with vehicle (Figure 6D).

### NR4A2 Over-expression Increases the Repair of UV Induced DNA Lesions

**Figure 7:**
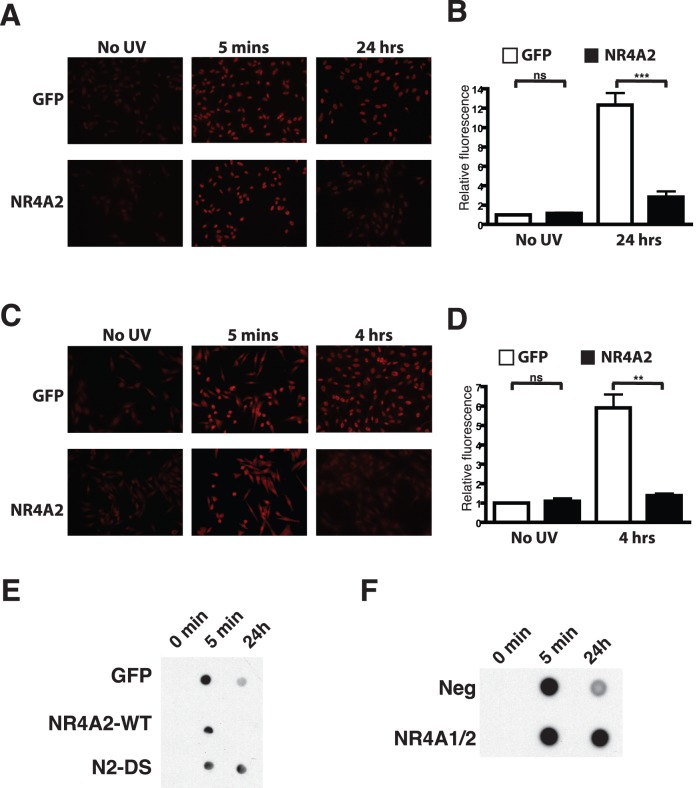
NR4A2 expression facilitates removal of UVR induced DNA lesions. (**A**) A2058 melanoma cell lines engineered to stably express either GFP or NR4A2 were subjected to mock or UVR treatment after which the levels of CPD’s were determined by immunofluorescence 24 hours post irradiation. Representative images are shown. (**B**) Quantification of CPD fluorescence of multiple images from three independent experiments is shown with values normalised to the A2058-GFP mock treated cells. Data is expressed as the mean +/− SEM of 3 independent experiments. (**C**) A2058-GFP and A2058-NR4A2 cells were treated as described above and 6-4-PP levels determined by immunofluorescence at 4 hours post irradiation. Representative images are shown. (**D**) Quantification of 6-4-PP fluorescence was performed as described above for the mock (no UV) and 4 hour post UVR conditions. Data is expressed as the mean +/− SEM of 3 independent experiments. (E) South-western blot analysis of CPD levels in A2058 cells stably expressing GFP, NR4A2-WT or N2-DS proteins following UVR treatment. (F) South-western blot analysis of CPD levels in MM96L cells, transfected with negative or pooled NR4A2 and NR4A1 siRNAs as indicated, following UVR treatment. In panels E and F, total genomic DNA was harvested prior to irradiation (0 min) and 5 minutes and 24 hours post irradiation as indicated.

We have previously reported the requirement for NR4A2 expression for the ability of the MC1R pathway to enhance the repair of UV induced CPD lesions in melanocytes [3]. This report together with our observation that the NR4A2 protein co-localises to distinct foci containing proteins involved in nucleotide excision repair prompted us to investigate the effect of NR4A2 expression on the removal of UV induced lesions. To achieve this we used a cutaneous melanoma cell line previously found to have low levels of NR4A1 and NR4A2 expression by real time PCR and EMSA. A2058 cells were engineered to express high levels of NR4A2 using lentiviral delivery and analysed in comparison to cells engineered using GFP expressing lentivirus. Polyclonal pools, obtained following several passages in antibiotic selection media were irradiated with 25 mJ/cm2 of UVR and harvested for immunofluorescence detection of CPDs (Figure 7A) or 6-4-PPs (Figure 7C) prior to irradiation (No UV), 5 minutes, and 4 hours (6-4-PPs) or 24 hours (CPDs) post irradiation. Quantification of the fluorescence confirmed a significant reduction of DNA lesions remained in the NR4A2 expressing cells compared to the GFP expressing cells at the indicated time-points (Figure 7B and 7D). CPD clearance was also tested using Southwestern-blotting of genomic DNA isolated from A2058 cells expressing GFP, NR4A2 or the N2-DS chimeric protein prior to irradiation and 5 minutes and 24 hours post UVR treatment (Figure 7E). These data also revealed a significantly enhanced clearance of CPD’s in cells over-expressing NR4A2 compared to the GFP control, while cells expressing the mutant chimera appeared to have impaired clearance rates. We next sought to determine if loss of NR4A expression would affect CPD clearance following UV irradiation. MM96L cells, which have been reported to express high levels of NR4A1 and NR4A2 were transfected with a negative or pooled NR4A1/NR4A2 siRNA, after which they were untreated or UV irradiated. South-western blotting of genomic DNA extracted from cells prior to, and 5 minutes and 24 hours post irradiation reveal a significant amount of CPD clearance in the negative siRNA transfected cells. While this clearance was less evident in the cells in which NR4A1/2 levels had been depleted (Figure 7F). Taken together, these observations suggest that gain or loss of NR4A2 expression can promote or impair CPD clearance respectively.

## Discussion

NR4As are a sub-family of nuclear receptors that co-ordinate gene expression programs underpinning neuronal development, inflammation and metabolism and are regulated by diverse range of signalling pathways. While primarily recognised as DNA binding transcriptional regulators, accumulating evidence is identifying novel roles, non-DNA binding/non-transcriptional, for these receptors [1], [2], [25]. We have previously demonstrated that these receptors facilitate the repair of UV induced DNA damage in the context of MC1R signalling in human melanocytes [3], however whether this was a direct or transcriptionally mediated effect was not determined. Accordingly we were prompted by these observations to explore the mechanism by which expression of the NR4A proteins facilitated clearance of UV induced DNA lesions. In the studies presented here we further demonstrate the rapid recruitment of EYFP-NR4A fusion proteins to novel nuclear foci in response to UV irradiation of cells. Co-localisation of proteins involved in the recognition and repair of DNA damage such as γH2A.X, and nucleotide excision repair proteins, XPC and DDB2, at these foci suggest these are sites of active DNA damage repair. While γH2A.X is widely recognised as a marker for double stranded DNA breaks, it’s role in the response to UV damage is less clear [26]. The induction of γH2A.X foci is frequently observed in response to UVR, however only a minority of these foci have been found to represent double stranded DNA breaks and most likely arise at sites of arrested replication forks [27], [28]. Localization studies performed here found that NR4A2 consistently co-localized with NER proteins XPC and DDB2, however only partially localised with γH2A.X suggesting a broader role for γH2A.X in response to UV damage. Such partial localisation has been observed previously between γH2A.X and 53BP1 in response to UVR [29]. In the context of DSB repair a recent report identified co-localisation of NR4A2 and γH2A.X in mouse embryonic fibroblasts following γ-irradiation and demonstrated that the recruitment of NR4A2 at sites of double stranded breaks was required for efficient repair of these lesions. This functionality was dependent on the interaction with DNA-PK and subsequent phosphorylation of the receptor in the DNA binding domain, however this phosphorylation event was not required for translocation of the receptor to double stranded breaks [20]. Here we report the requirement of p38 activity for the recruitment of the NR4A proteins to distinct nuclear foci in response to UVR. Moreover, truncations and point mutations of the N-terminal domain suggest that direct phosphorylation of this region of the NR4A2 protein is required for recruitment of the receptor to these sites. The observation that the NTD alone is sufficient to promote UV induced translocation to foci would argue for a protein:protein interaction that is specific to the NTD of NR4A2, a contention further supported by the ability of a chimeric receptor containing the NR4A2 N-terminal domain and the PPARγ DNA and ligand binding domain to be recruited to DNA damage foci, a function not performed by the full length PPARγ protein. This observation is consistent with a specific protein interaction by the NTD, which is facilitated by direct phosphorylation by p38 initiating the translocation of these proteins to sites of DNA damage recognition and repair. Phosphorylation and activation of p38 has also been found to be crucial for recruitment of early sensors of DNA damage such as DDB2 and XPC [30], [31].

UVR is one of the most common exogenous mutagens encountered by human populations and a direct association between lifetime UVR exposure and the development of melanoma and non-melanoma skin cancer has long been recognised [32]. Complex cellular mechanisms have evolved to identify, process and repair different types of DNA damage. In the context of UV radiation the most common genetic lesions arise from CPD and 6-4PPs, which are removed by the process of Nucleotide Excision Repair (NER). NER and other DNA repair mechanisms are comprised of large multi-protein complexes that are recruited to, and removed from, sites of DNA damage in a timely and sequentially ordered fashion [5]. In order for such large protein complexes to assemble and function at sites of DNA damage the physical barrier posed by the chromatin environment surrounding the lesion must be overcome. Direct involvement of NR4A2 in facilitating the repair of double stranded breaks induced by gamma irradiation [20] taken together with our data demonstrating co-localisation with NER machinery and participation in clearance of UV induced lesions by NER argues for a general role for the NR4A family in the detection and response to different types of DNA damage. The modulation of the chromatin environment by nuclear receptors in the context of transcriptional regulation is well established [21], [22] and similar chromatin rearrangements are known to be crucial for efficient DNA repair [33], [34]. Indeed it has been reported that the enzymes that co-ordinate the excision and re-ligation of DNA lesions require a minimum of 100 bp of DNA flanking the lesion for efficient repair [35]. Interestingly, p38 kinase activity is known to promote chromatin relaxation with a concomitant enhancement of NER [36]. The results presented here support a direct role for p38 in the recruitment of the NR4A proteins to DNA damage foci and given the potential ability for nuclear receptors to facilitate chromatin rearrangements it is plausible that recruitment of these proteins contributes to p38 mediated chromatin relaxation.

PARP-1 is known to play key roles in cellular responses to genotoxic stress and in a number of DNA repair pathways such as double stranded break repair (Reviewed in [37]). The recognition that recruitment of NR4A2 to double stranded DNA breaks was dependent on PARP1 activity [20] prompted us to examine if a similar functional requirement is also required for formation of NR4A2 containing foci in response to UV irradiation. This is indeed the case and co-immunoprecipitation assays suggest a direct physical interaction between NR4A2 and PARP1. UV induced DNA damage is known to activate PARP1 [38] and more recent studies have demonstrated that this activity promotes NER by stabilizing the DDB2 component of the NER machinery [39]. In the context of NER PARP1 activity appears to facilitate improved interactions between DDB2 and other NER proteins such as XPC [40] and co-operates with DDB2 in mediating a more relaxed, and hence accessible chromatin environment to promote improved DNA repair [41]. It is plausible to propose that interaction between PARP1 and NR4A2, in addition to PARP1 activity, may play a similar role in stabilizing interactions between NR4A2 and other yet to be identified DNA repair proteins. Whether NR4A2 is directly ADP-ribosylated by PARP1 in response to UVR remains to be determined, however, the activity of PARP1 is essential for NR4A2 foci formation following UVR. Given the well established role of nuclear receptor proteins in modulating the chromatin environment in the context of transcriptional regulation, and the functional link between PARP-1 and DDB2 in facilitating chromatin accessibility for NER, it is possible that a PARP1-NR4A2 functional co-operation may have a similar impact on chromatin relaxation to augment recognition and repair of UV induced DNA lesions.

Here we have utilised mutant forms of NR4A2 that would be expected to alter co-regulator recruitment and ultimately alter chromatin rearrangements. Our data suggests that disrupting the functional integrity of the receptor can have deleterious consequences on the co-localisation the NR4A proteins with other DNA repair proteins. While DNA repair proteins XPC, DDB2 and γH2A.X, and the NR4A proteins still respond to UVR exposure by forming distinct nuclear foci, the mutant NR4A2 proteins rarely, if ever, co-localise to the same foci. It is possible that recruitment of the mutant form of NR4A2 to DNA damage foci may exclude the DNA repair machinery as a consequence of altered chromatin dynamics. The ability of the NR4A2-ΔH12 and N2-DS mutant proteins to exclude the XPC, DDB2 and γH2A.X proteins suggests that NR4A recruitment is one of the early events associated with foci formation, perhaps preceding recruitment of the repair machinery. Moreover, the re-established co-localisation of repair proteins with the mutant NR4A proteins in the presence of histone deacetylase inhibitors further supports the concept that the presence of NR4A proteins at repair foci is an early event. While such foci are commonly observed in response to γ-irradiation and interpreted to represent double stranded DNA breaks, accumulating evidence suggests such foci are indicative of architectural alterations of chromatin formed by numerous forms of DNA damage [42].

Co-localisation with repair machinery and the demonstration that over expression of NR4A2 can increase clearance of UVR induced DNA lesions represents the first evidence for direct involvement of the NR4A receptors in the NER process. These observations coupled with recent reports demonstrating NR4A2 mediated repair of double stranded breaks following γ-irradiation argue for a broad role for the NR4A proteins in mediating repair of multiple types of DNA lesions. Previous reports using NR4A1/NR4A3 double knock-out mice revealed a striking phenotype of early postnatal lethality arising from the development of acute myeloid leukaemia [43]. More recent interrogation of this model has confirmed that NR4A gene dosage leads to myloid neoplasms and the identification of alterations in DNA damage check-point proteins lends further support for NR4A mediated genome protection [44]. While different mechanisms are employed by cells to repair DNA damage dependent on the type of lesion, the transcriptional activity at the damage loci and cell cycle status of the cell when damage is incurred, the physical barrier posed by the chromatin environment surrounding the damage is a unifying feature that must be addressed in all contexts. Finally our results afford novel insights into the process of UV induced skin carcinogenesis and highlight a role for the NR4A proteins in maintaining genomic integrity following mutagenic insults, with any disruption of these receptors having direct relevance to cancer formation.

## Materials and Methods

### Plasmid Constructs

The NR4A2 open reading frame was PCR amplified using NR4A2-Fwd: 5′-TCTCGAGCTCAAGCTTCGATGCCTTGTGTT-3′ and NR4A2-Rev: 5′-GATCCGGTGGATYCCCTTAGAAAGGTAAGGT-3′ primers and cloned into the HindIII and XbaI sites of the pEYFP-C1 vector (Clontech). The pEYFP-NR4A2-ΔH12 deletion mutant was generated by PCR using the NR4A2-Fwd and NR4A2-ΔH12-Rev: 5′- GCGCTCTAGATTATACCAAGTCTTCCAATTT-3′ primers and cloned as described above. The pEYFP-NR4A2 clone was NR4A2 point mutations were generated from dual PCR products (ATG to mutation, and mutation to Stop codon) using forward or reverse primers to engineer the nucleotide change required for the corresponding amino-acid change. Dual PCR products were recombined using the In-Fusion PCR cloning system as per manufacturers instruction (Clontech). Similarly, the N2-DS NR4A2- PPARγ fusion construct was generated by engineering PCR products covering the NR4A2 NTD and the DBD to LBD region of a dominant negative PPARγ gene, subsequently stitched together using the In-fusion enzyme (Clontech). GFP and NR4A2 open reading frames were cloned into the pLVX-Puro lentiviral construct as per manufacturers instruction (Clontech). Primer sequences available upon request.

### Cell Culture and Transfection Analysis

The human melanoma cell lines MM96L [11] and A2058 (ATCC, Cat. No. CRL-11147) were cultured in RPMI media supplemented with 10% foetal bovine serum, 2 mM L-glutamine, and 5 mg/ml penicillin/streptomycin. Drug treatments using H89, GF109203X, PD98059, SB203580 (Sigma Aldrich, Sydney, NSW, Australia) and PJ34 (Santa Cruz Biotechnology In.) were all performed at a final concentration of 10 µM or an equivalent volume of DMSO (vehicle) control were added to media 10 minutes prior to mock or UVR treatment. Trichostatin A (Sigma Aldrich, Sydney, NSW, Australia) or ethanol (vehicle) control were added to media to a final concentration of 100 mM immediately after UVR treatment. For fluorescence studies cells were grown in 12 well tissue culture dishes on glass coverslips after which they were transfected with plasmids as indicated in results section using Lipofectamine-2000 (Invitrogen) as per manufacturers recommendations. NR4A1/2 siRNA transfection in MM96L cells was performed in 6 well dishes as previously described [3], [12].

### Lentivirus Transduction

GFP and NR4A2 lentivirus was generated using the Lenti-X HT packaging system (Clontech) according to the manufacturers protocol. For maximum transduction efficiency, A2058 melanoma cells were treated with media containing harvested lentiviral particles with 4 mg/ml polybrene and centrifuged at 32°C at 2, 830 RPM for 90 minutes. Fresh media was replaced at 24 hours post-centrifugation. Cells were then selected in media containing 1 ug/ml puromycin prior to performing experiments.

### UV Treatment and Fluorescent Imaging

Cells transfected on coverslips were irradiated with 25 mJ/cm^2^ as previously described [3]. For analysis of foci formation cells were fixed 4 hours post irradiation. Immunofluorescence was performed as previously described [3]. Briefly, cells were then permeabilized in 0.1% Triton X-100 diluted in PBS and blocked in immunofluorescence buffer with 0.5% BSA diluted in PBS. Primary antibodies use were diluted in 0.5% BSA. 1∶9 dilution for mouse monoclonal anti-DDB2 (2246C4a, abcam); 1∶400 dilution for rabbit polyclonal anti-XPC (A301-122A, BETHYL Laboratories); and 1∶400 dilution for mouse monoclonal anti-γH2A.X (MA1-2022, Pierce antibodies). Alexa Fluor 594-conjugated secondary antibodies, donkey anti-mouse and donkey anti-rabbit were used at a 1∶200 dilution. Nuclei were then counterstained with DAPI and mounted as previously described. Stable A2058 cells expressing GFP or NR4A2 were irradiated and fixed in 4% paraformaldehyde 5 minutes, 4 hours or 24 hours post irradiation. Immunofluorescence of CPD and 6-4-PP lesions was performed as previously described [3] using anti-cyclobtane pyrimidine dimer (TDM-2; MBL International Corp.) or anti-(6-4) photoproducts (64-M2; MBL International Corp.) antibodies according to the manufacturer’s instructions. Images were obtained using an Olympus BX-51 epifluorescence microscope and a DP-70 camera. Fluorescence intensity was quantified on a BD-Pathway 855 High-Content-Screening platform using AttoVision 1.5 software according to the manufacturer’s instructions.

### Southwestern Blot Analysis of CPD

A2058 cells overexpressing GFP, NR4A2 or N2-DS chimera proteins, were plated into 6 well dished and grown to 70% confluency. Cells were UV irradiated as described above and harvested prior to UV irradiation or 5 minutes or 24 hours post irradiation. Total genomic DNA was harvested using a Qiagen DNeasy Blood and Tissue DNA extraction kit (Qiagen). 100 ng of total genomic DNA for each condition was transferred to a nylon filter and probed for CPD levels using anti-cyclobtane pyrimidine dimer (TDM-2; MBL International Corp.) as described previously [13]. Southwestern blot analysis as described above was also performed using MM96L cells transfected with negative or NR4A1 and NR4A2 siRNA.

### Statistical Analysis

Statistical analyses were performed using GraphPad-PRISM software based on a minimum of three independent experiments using an unpaired student *t* test or one-way ANOVA followed by a Tukey’s post-test. Significance was scored as: ***, p<0.0001; **, p<0.001; *, p<0.05; ns, not significant.

## Supporting Information

Figure S1 **NR4A2-** **PPARγ dom neg chimera, N2-DS, is recruited to nuclear foci but fails to localise with DNA repair proteins.** (**A–C**) MM96L cells transfected with WT NR4A2 or the N2-DS chimera were treated with 25 mJ/cm2 UV, fixed at 4 hours post-UV treatment. Immunofluorescence was performed to detect (**A**) γH2A.X; (**B**) DDB2; and (**C**) XPC proteins. Left panels (green fluorescence), pEYFP-NR4A-WT (upper) or pEYFP-N2-DS (lower); Middle panels, γH2A.X, DDB2 or XPC immunofluorescence (red); Right panels, merged images; Representative images from 4 independent experiments is shown.
(EPS)

Figure S2 **Trichostatin A (TSA) treatment restores co-localisation of N2-DS chimera and DNA repair proteins.** The co-localisation of the N2-DS chimera (green) and (**A**) γH2A.X, (**B**) DDB2 or (**C**) XPC shown in red was performed in the presence of vehicle (upper panels) or TSA (lower panel). These data are representative images from 4 independent experiments. Left panels (green fluorescence), pEYFP-NR4A2-N2-DS; Middle panels, γH2A.X, DB2 or XPC immunofluorescence (red); Right panels, merged images; Representative images from 4 independent experiments is shown.
(EPS)

Figure S3 **Trichostatin A (TSA) treatment restores the transcriptional activity of NR4A2-ΔH12.** (A) Schematic representation of pNBRE-tk_LUC transiently transfected into COS-1 cells with WT or ΔH12 mutant form of NR4A2. (B) Transcriptional activity of empty pEYFP vector, pEYFP-NR4A2-WT and pEYFP-NR4A2-ΔH12 in the presence of Vehicle or TSA. Each data point represents the mean±SEM of results from 3 independent experiments. Statistical analysis was performed using One-way ANOVA with a Tukey’s post-test (**, *p*<0.001; *, *p*<0.01; *ns,* not significant).
(EPS)
